# Relative predictive value of lung cancer screening CT versus myocardial perfusion attenuation correction CT in the evaluation of coronary calcium

**DOI:** 10.1371/journal.pone.0175678

**Published:** 2017-04-24

**Authors:** Grant Bailey, Abigail Healy, Bryan D. Young, Esseim Sharma, Judith Meadows, Hyung J. Chun, Wen-Chih Wu, Gaurav Choudhary, Alan R. Morrison

**Affiliations:** 1 Department of Internal Medicine (Section of Cardiovascular Medicine), VA Connecticut Healthcare System, West Haven, Connecticut, United States of America; 2 Department of Internal Medicine (Section of Cardiovascular Medicine), Yale Cardiovascular Research Center, Yale University School of Medicine, New Haven, Connecticut, United States of America; 3 Department of Internal Medicine (Section of Cardiovascular Medicine), Providence VA Medical Center, Providence, Rhode Island, United States of America; 4 Department of Internal Medicine (Section of Cardiovascular Medicine), Alpert Medical School of Brown University, Providence, Rhode Island, United States of America; Nagoya University, JAPAN

## Abstract

Coronary artery calcium scores (CACS) from lung cancer screening computed tomography (LCSCT) or myocardial perfusion attenuation correction computed tomography (ACCT) are not routinely performed or reported. CACS from LCSCT and ACCT have not been directly compared in the same patient population. We identified 66 patients who underwent both LCSCT (non-gated) and ECG-gated cardiac CT (CCT) within a 2-year span. Of this population, 40 subjects had also undergone ACCT. Using the Agatston method, CACS for 264 individual vessels from the LCSCT population and for 160 vessels from ACCT population were calculated and evaluated for agreement with ECG-gated CCT as the gold standard. Secondary analysis included a comparison of individual vessel contribution to variations in agreement and a comparison of total CACS from CCT, LCSCT, and ACCT for respective MACE prediction. CACS from LCSCT demonstrated a strong Pearson correlation, *r* = 0.9017 (0.876–0.9223), with good agreement when compared to CACS from CCT. CACS from ACCT demonstrated a significantly (P < 0.00001) weaker correlation, *r* = 0.5593 (0.4401–0.6592). On an individual vessel basis, CACS from all major vessels (LM, LAD, LCX, and RCA) contributed to the weaker correlation. For total vessel CACS, LCSCT demonstrated comparable area under the curve (AUC) for the receiver operating characteristic (ROC) curve (LCSCT AUC = 0.8133 and CCT AUC = 0.8302, P = 0.691) for prediction of MACE. Although ACCT demonstrated a similar AUC (ACCT AUC = 0.7969, P = 0.662) for MACE prediction the cutoff value for elevated risk was extremely low. In conclusion, LCSCT outperformed ACCT at calcium scoring by providing better agreement and comparable risk assessment to CCT despite the absence of ECG-gating. It is therefore reasonable to use LCSCT images to derive and report Agatston-based CACS for cardiovascular risk assessment, whereas the use of ACCT images to report Agatston-based CACS is not currently practical.

## Introduction

According to the World Health Organization, ischemic heart disease caused by atherosclerotic coronary artery disease (CAD) remains the single leading cause of morbidity and mortality in the world[1]. Calcification of atherosclerotic plaques is a well-established pathologic finding that carries predictive value in terms of atherosclerotic burden and risk of cardiovascular mortality and all-cause mortality[2, 3]. In addition, the organization of calcific deposits within individual atherosclerotic plaque has predictive value for the plaque’s vulnerability[4–6]. ECG-gated CCT defines coronary calcium by a threshold attenuation coefficient measurement of 130 Hounsfield units (HU) with an area of ≥1 mm^2^, and CACS are then determined by the product of calcified plaque area and relative density as determined by attenuation[7]. Other large epidemiologic studies have used a threshold of 130 HU with an area of ≥1.48 mm^2^ to identify coronary calcium[8, 9]. In line with the goals of precision medicine, the addition of coronary artery calcium score to prediction models based on traditional risk factors significantly improves risk stratification and can place more individuals in the most extreme risk categories, thus having potential to influence decision making with regards to medical therapy[10, 11].

Lung cancer, the third most common cancer and leading cause of cancer-related death, can be treated with surgical resection which is potentially curative in early stages[12, 13]. Low-dose, non-gated, noncontrast chest CT (LCSCT) scans have provided a significant benefit for lung cancer screening in the high-risk smoking population and are recommended for routine cancer screening[13–17]. Because smoking is also a major risk factor for ischemic heart disease and there is significant overlap between eligibility for LCSCT and elevated cardiovascular risk, there is broad interest in using information about coronary calcification from LCSCT to aide in cardiovascular event risk assessment[18–21]. In the evaluation of myocardial ischemia, hybrid imaging using either single-photon emission computed tomography (SPECT) or positron emission tomography (PET) combined with CT allows for improved diagnostic accuracy of myocardial perfusion imaging through the use of CT-based attenuation correction algorithms[22–26]. Moreover, there may be additional benefit to assessing coronary calcium on attenuation CT scans during myocardial perfusion imaging to help further predict cardiovascular event risk[27–29].

While studies have demonstrated reasonable agreement between CACS from various types of non-gated, noncontrast chest CT and ECG-gated CCT, clinical outcomes have rarely been evaluated[30–34]. Conversely, recent studies that demonstrated the predictive value of CACS from LCSCT did not directly compare LCSCT with the gold standard, ECG-gated CCT, for agreement[19, 20]. Moreover, despite showing strong potential for providing information about coronary artery calcium burden, there has been no direct comparison of ACCT with CCT in terms of Agatston CACS values or MACE prediction[27–29]. Lastly, there have been no studies that directly compare both LCSCT and ACCT with CCT in the same population.

Here, we identified a patient population who had undergone LCSCT, ACCT, and CCT within a 2-year period. We evaluated agreement and predictive value of Agatston-based CACS from LCSCT and ACCT scans compared to CCT as a gold standard. To our knowledge, this is the first direct evaluation of both LCSCT and ACCT relative to CCT for the prediction of outcomes in an elevated risk population, using a standard Agatston-based scoring method.

## Materials and methods

This study was approved by the VA Connecticut Healthcare Institutional Review Board and complies with the Declaration of Helsinki, and all patient data were handled in compliance with the Health Insurance Portability and Accountability Act (HIPAA) regulations. All patient records were de-identified and analyzed anonymously.

### Study design and patient population

We undertook a retrospective study design. This study was performed at a single site, the Veterans Affairs (VA) Healthcare System Medical Center in West Haven, Connecticut. This study included all U.S. Veterans who were identified as having undergone both LCSCT and CCT within a 2-year period between October 1, 2012 and September 30, 2015. We identified 66 patients that met these criteria. Of the 66 patients, 40 patients had also undergone a myocardial perfusion imaging study with ACCT within the same 2-year period.

### CT acquisition and reconstruction parameters

Table 1 summarizes all CT Acquisition and Image Reconstruction Parameters.

**Table 1:** CT acquisition and image reconstruction parameters.

	CCT	LCSCT	ACCT
Collimation	64 x 0.5 mm	64 x 0.5 mm	16 x 1.5 mm
Acquisition Protocol	Axial	Helical	Helical
Rotation Time	0.23	0.35–0.4	0.4
Pitch	N/A	0.84	0.81
FOV	320	400	600
Matrix Size	256 x 256	256 x 256	512 x 512
Area Required to Identify Calcium	≥1.56 mm^2^	≥2.44 mm^2^	≥1.37 mm^2^
Tube Voltage	120 kVp	100 kVp	120 kVp
Tube Current	73 mAmp	50–70 mAmp	30 mAmp
Image Reconstruction Slice Thickness	3.0 mm	2.0 mm	5.0 mm
Average Slices Containing the Heart, mean ± s.d. (P value)	32 ± 5.5 (<0.0001)	44 ± 5.1 (<0.0001)	22 ± 4.9 (<0.0001)

P value relative to other CT values, as determined by ordinary one-way ANOVA followed by Tukey’s post hoc multiple comparisons test.

#### 1. CCT

Studies at the West Haven VA Medical Center were performed using a 64-slice CT scanner (Toshiba Acquilion) with a 64 mm x 0.5 mm collimation, using an axial acquisition protocol. Rotation time was 0.23 seconds. All scans were ECG-gated and electrocardiographically triggered at 70% of the R-R interval, with patient heart rates averaging between 55–65 BPM. Scanning field of view was set to 320 mm. Matrix size was 256 x 256. Based on these parameters, the minimum area required to identify calcium was 1.56 mm^2^. A 120-kV tube voltage was applied for all subjects. The tube current was 73 mAmp. Image reconstruction slice thickness was 3 mm.

#### 2. LCSCT

Studies at the West Haven VA Medical Center were performed using a 64-slice CT scanner (Toshiba Acquilion) with a 64 mm x 0.5 mm collimation, using a helical acquisition protocol. Rotation time was 0.35–0.4 seconds, and pitch was 0.84. Studies were not ECG-gated, and patient heart rates were not controlled. Scanning field of view was set to 400 mm. Matrix size was 256 x 256. Based on these parameters, the minimum area required to identify calcium was 2.44 mm^2^. The average tube voltage was 100 kV, and the tube current was modulated between 50–70 mAmp. Image reconstruction slice thickness was 2 mm.

#### 3. ACCT

Studies at the West Haven VA Medical Center were performed using a 16-slice CT scanner (Phillips Precedence) with a 16 mm x 1.5 mm collimation, using a helical acquisition protocol. Rotation time was 0.4 seconds, and pitch was 0.81. Studies were not ECG-gated, and patient heart rates were not controlled. Scanning field of view was set to 600 mm. Matrix size was 512 x 512. Based on these parameters, the minimum area required to identify calcium was 1.37 mm^2^. A 120-kV tube voltage was applied for all subjects. The tube current was 30 mAmp. Image reconstruction slice thickness was 5 mm.

### Coronary artery calcium scoring

For CCT, LCSCT, and ACCT scans, CACS was calculated using the Agatston method[7]. Coronary artery calcium scoring was performed using previously described methods for other epidemiologic studies[8, 9], and images were viewed and scored using a Carestream Vue PACS (Carestream Health) imaging workstation. In brief, the calcium scoring application in the Carestream Vue PACS displays axial image slices for the reader. The calcium scoring application was equally successful at presenting axial images from CCT, LCSCT, and ACCT. As expected, the mean numbers of slices taken up by the heart on axial images were statistically different 32 ± 5.5, 44 ± 5.1, and 22 ± 4.9 for CCT, LCSCT, and ACCT studies, respectively (see Table 1). The reader scrolls through axial slices and identifies coronary arteries with potential calcium to be scored. The reader indicates the appropriate coronary vessel to the program and circles the region of interest. Only pixels of attenuation coefficient measurements above 130 HU within the region of interest are selected as calcified and incorporated into the calcium score. All scans were scored twice; once by a board-certified cardiologist in Cardiovascular Computed Tomography (A.R.M. and J.M) and once by a cardiac imaging residents and fellows (G.B., E.S., and B.D.Y.). Residents and fellows were trained on 50 scans with feedback and supervision from the board-certified cardiologists. All readers were blinded to patient clinical data and the calcium scores from the different CT modalities (CCT, LCSCT, and ACCT). Inter-observer agreement was quantified for total CACS values from all (CCT, LCSCT, and ACCT) studies, and the overall kappa was found to be very good at 0.915 with 95% confidence interval from 0.867 to 0.964.

### Covariates

The electronic medical record was searched for patient demographics and cardiovascular covariates including age, sex, race, and BMI. Medical history included smoking status, hypertension, hypertensive medication, cholesterol medication, diabetes and diabetes medication, and fasting lipid profile. 10-Year Framingham risk score and ASCVD risk score were calculated.

### Outcomes

The electronic medical record was searched for major adverse cardiovascular events (MACE) as defined by death by all cause, sudden cardiovascular death, death by cerebrovascular accident, nonfatal cerebrovascular accident, nonfatal myocardial infarction, acute coronary syndrome, and revascularization by either percutaneous intervention (PCI) or coronary artery bypass graft surgery (CABG).

### Statistical analysis

All statistical data were analyzed with the use of Prism 6 (GraphPad) or R software version 3.3.1 (R Core Team). Baseline demographics, clinical characteristics, and CACS were compared between the cohort that underwent LCSCT and the subgroup who also underwent ACCT. Results are presented as mean (standard error) for continuous variables with normal distribution, as median (interquartile range) for continuous variables without normal distribution, and as number (percentage) for categorical data. The *t* test was used to compare normally distributed continuous variables between 2 independent groups. The Wilcoxon rank sum test was used for continuous variables not normally distributed, and Chi-square test was used for categorical variables. Differences between multiple groups were assessed by ANOVA followed by Tukey’s post hoc multiple comparisons test. For analysis of CACS between LCSCT and CCT or ACCT and CCT, the Pearson product-moment correlation coefficient was determined followed by a Bland-Altman plot for bias and agreement. Analyses were repeated using Generalized Estimating Equations (GEE) to adjust for potential clustering of coronary vessel’s calcium score by patient. For receiver operating characteristic curves, comparison of the areas under the curve between LCSCT and CCT as well as between ACCT and CCT were carried out by the method established by Hanley and McNeil[35]. Time-to-event curves using the Kaplan-Meier method were calculated. Results were compared using the log-rank statistic. A 2-sided *P*<0.05 was considered statistically significant. Using a two-sided alpha of 0.05, our study had an 80% power to detect a 10% difference in the Pearson correlation between LCSCT and ACCT. Using a two-sided alpha of 0.05, our study had an 80% power to detect significance in the proportion of events using calcium score cutoff for CCT.

## Results

We evaluated a total of 66 patients who had undergone both LCSCT and CCT within the period of 3 years between October 1, 2012 and September 30, 2015 (Table 2). The 66 patients in this study had a mean age of 65 years. All patients were U.S. Veterans. A majority of the patients were white men and smokers with elevated cardiovascular risk. The median cholesterol was 169 mg/dL and the median BMI was 31 kg/m^2^. Approximately one-third of the patients carried a history of diabetes or a family history of early CAD. Fifteen percent of patients had known CAD by a prior imaging modality, but none of the patients had a previous history of revascularization. Of the 66 patients, 40 patients had also undergone myocardial perfusion imaging with ACCT. There were no differences in the demographics and clinical characteristics between the patients who underwent LCSCT and the patients who underwent both LCSCT and ACCT (Table 2). The median CACS as determined by CCT from both LCSCT and the ACCT populations was comparable at 160 and 176 (P = 0.731), respectively. The median time between CCT and non-gated CT was 7 (IQR: 1,17) months for LCSCT and 2 (IQR: 1,5) months for ACCT.

**Table 2:** Baseline demographics, clinical characteristics, and CACS of LCSCT vs. ACCT populations.

	LCSCT (*n* = 66)	ACCT (*n* = 40)	P Value
Age, years, median (IQR)	65 (58, 67)	65 (59, 67)	0.919
BMI, median (IQR)	31 (26, 35)	31 (27, 34)	0.987
Male, *n* (%)	61 (92)	37 (93)	0.715
Caucasian, *n* (%)	57 (86)	34 (85)	0.927
African American, *n* (%)	9 (14)	6 (15)	0.927
DM, *n* (%)	19 (29)	12 (30)	0.931
Hypertension, *n* (%)	49 (74)	29 (73)	0.976
Hyperlipidemia, *n* (%)	43 (65)	31 (78)	0.261
Total Cholesterol, median (IQR)	169 (145, 201)	171 (147, 204)	0.825
HDL Cholesterol, median (IQR)	36 (36, 55)	41 (36, 54)	0.759
Statin Use, *n* (%)	39 (59)	29 (73)	0.235
Smoking, *n* (%)	51 (77)	29 (73)	0.748
Family History of Early CAD, *n* (%)	20 (30)	16 (40)	0.487
CAD, *n* (%)	10 (15)	9 (23)	0.487
MI, *n* (%)	1 (2)	1 (3)	0.708
Prior PCI or CABG, *n* (%)	0 (0)	0(0)	N/A
Framingham Risk, median (IQR)	16 (12, 19)	16 (13, 18)	0.809
ASCVD Risk, median (IQR)	22 (15, 28)	21 (16, 28)	0.868
CCT CACS, median (IQR)	160 (14, 441)	176 (18, 500)	0.731

BMI = body mass index; DM = diabetes mellitus; CAD = coronary artery disease; MI = myocardial infarction; PCI = percutaneous intervention; CABG = coronary artery bypass graft surgery; ASCVD = atherosclerotic cardiovascular disease; CT = computed tomography; CACS = coronary artery calcium score; AC = attenuation correction

**Fig 1:**
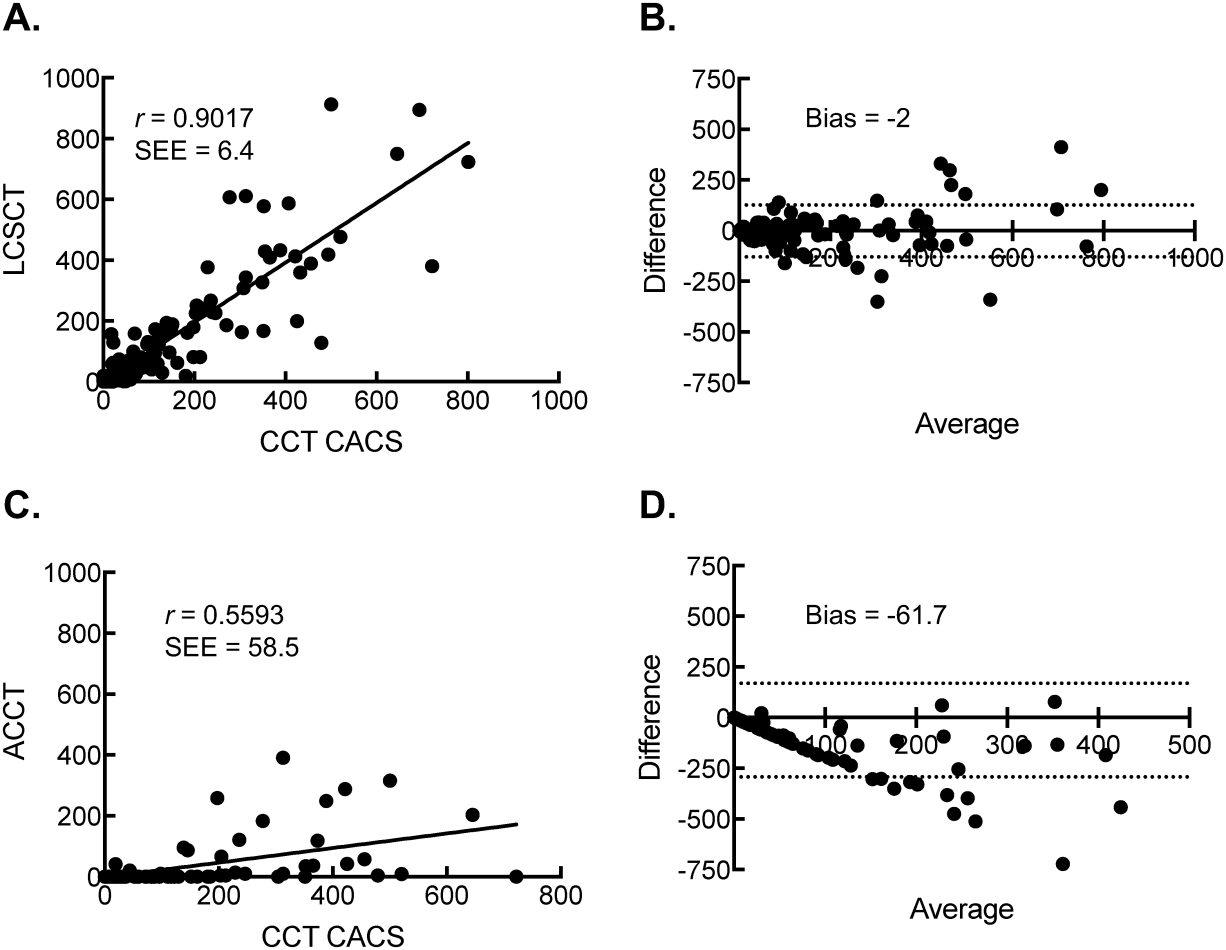
Scatter plot of Agatston CACS from CCT scans and either LCSCT or ACCT scans along with corresponding Bland-Altman plots for agreement. (A) The Pearson correlation of CACS between CCT scans and LCSCT scans. (B) Bland-Altman Plots for Agreement between CCT scans and LCSCT scans. (C) The Pearson correlation of global CACS between CCT scans and ACCT scans. (D) Bland-Altman Plots for Agreement between CCT scans and ACCT scans.

The agreement of individual coronary artery CACS between either LCSCT (264 coronary arteries) or ACCT (160 coronary arteries) and CCT were evaluated (Fig 1). Despite the lack of ECG-gating, the Pearson correlation between CACS from the CCT and the CACS from LCSCT was strong at 0.9017 (SEE = 6.4, P < 0.0001). Bland-Altman analysis showed a mean bias of -2 with 95% limits of agreement between -130 to 127. The Pearson correlation between CACS from the CCT and the CACS from ACCT was 0.5593 (SEE = 58.5, P < 0.0001). The Bland-Altman analysis showed a mean bias of -61.7 with 95% limits of agreement between -293 to 169. The Pearson correlations were statistically different between the LCSCT and the ACCT CACS (P < 0.00001). Results did not significantly change after adjusting for potential clustering effect at the individual patient level.

**Fig 2:**
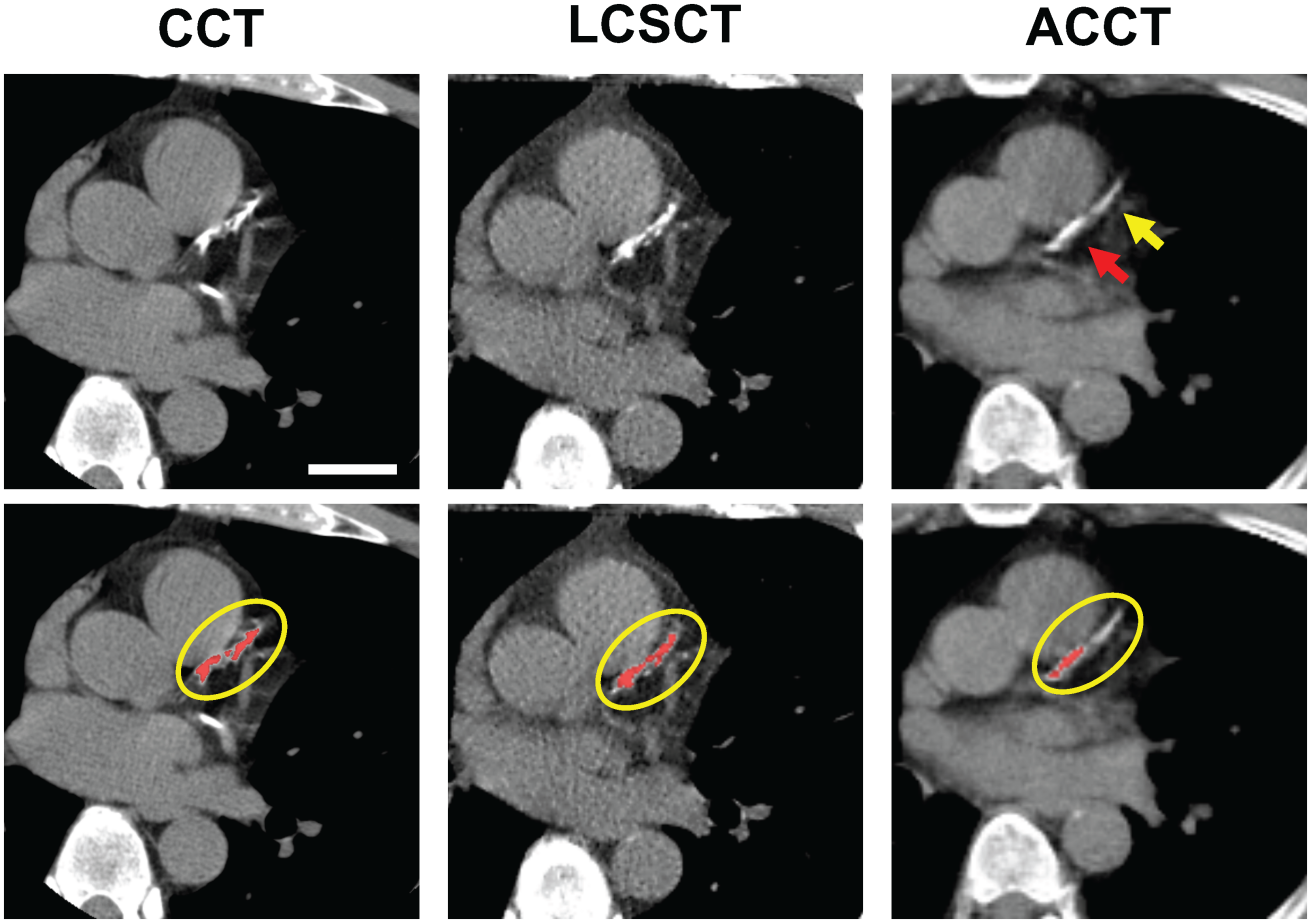
Example of the decreased detection of calcium using ACCT relative to CCT and LCSCT in a single slice plane of the LAD from an individual patient. CCT with LAD Agatston CACS of 645. LCSCT with LAD Agatston CACS of 749. ACCT with LAD Agatston CACS of 203. Bar, 3cm. Upper panel; CT images at the level of the proximal to mid LAD. Red arrow; identified calcium. Yellow arrow; unidentified calcium. Lower panels; CT images as displayed at the imaging workstation with pixels of Agatston threshold of 130 Hounsfield units (HU) highlighted in red and region of interest circled in yellow. In this example, LCSCT images were acquired 14 months after the CCT images and the ACCT images were acquired 3 months prior to the CCT images.

We assessed whether any individual vessel calcium scores contributed more or less to the significantly decreased agreement of ACCT-derived CACS (Table 3). The CACS were divided by individual vessel and then each vessel CACS was compared for agreement with the respective CACS from CCT studies as the gold standard. The Pearson correlations for the LCSCT studies were 0.9599 (0.9349–0.9754), 0.929 (0.8854–0.9564), 0.9169 (0.8668–0.9486), and 0.8548 (0.7702–0.9099) for left main (LM), left anterior descending (LAD), left circumflex (LCX), and right (RCA) coronary arteries, respectively. The Pearson correlations for the ACCT studies were 0.8424 (0.7176–0.9148), 0.5134 (0.2272–0.718), 0.7246 (0.5304–0.8466), and 0.5393 (0.2696–0.7305) for LM, LAD, LCX, and RCA respectively. On an individual vessel basis, the ACCT demonstrated decreased agreement relative to LSCST for all four major vessels, indicating that the weaker correlation is global and not particular to any specific vessel, nor attributable to the lack of ECG-gating or motion artifact of any one vessel. Fig 2 demonstrates an example of the discrepancy in agreement between images from ACCT and images from both LCSCT and CCT with areas of visually appreciable calcium in the LAD that fell below the Agatston threshold of 130 HU on the ACCT image (ACCT, upper panel, yellow arrow), leading to an under-identification of the calcium burden in the ACCT study.

**Table 3:** Comparison of the Pearson correlation of total CACS and individual vessel CACS between LCSCT and ACCT.

Vessel	LCSCT *r* (95% Cl)	ACCT *r* (95% CI)	P Value
Total	0.9385 (0.9007–0.9621)	0.6204 (0.3751–0.7845)	< 0.00001
LM	0.9599 (0.9349–0.9754)	0.8424 (0.7176–0.9148)	0.0006
LAD	0.929 (0.8854–0.9564)	0.5134 (0.2272–0.718)	< 0.00001
LCX	0.9169 (0.8668–0.9486)	0.7246 (0.5304–0.8466)	0.0016
RCA	0.8548 (0.7702–0.9099)	0.5393 (0.2696–0.7305)	0.0012

**Fig 3:**
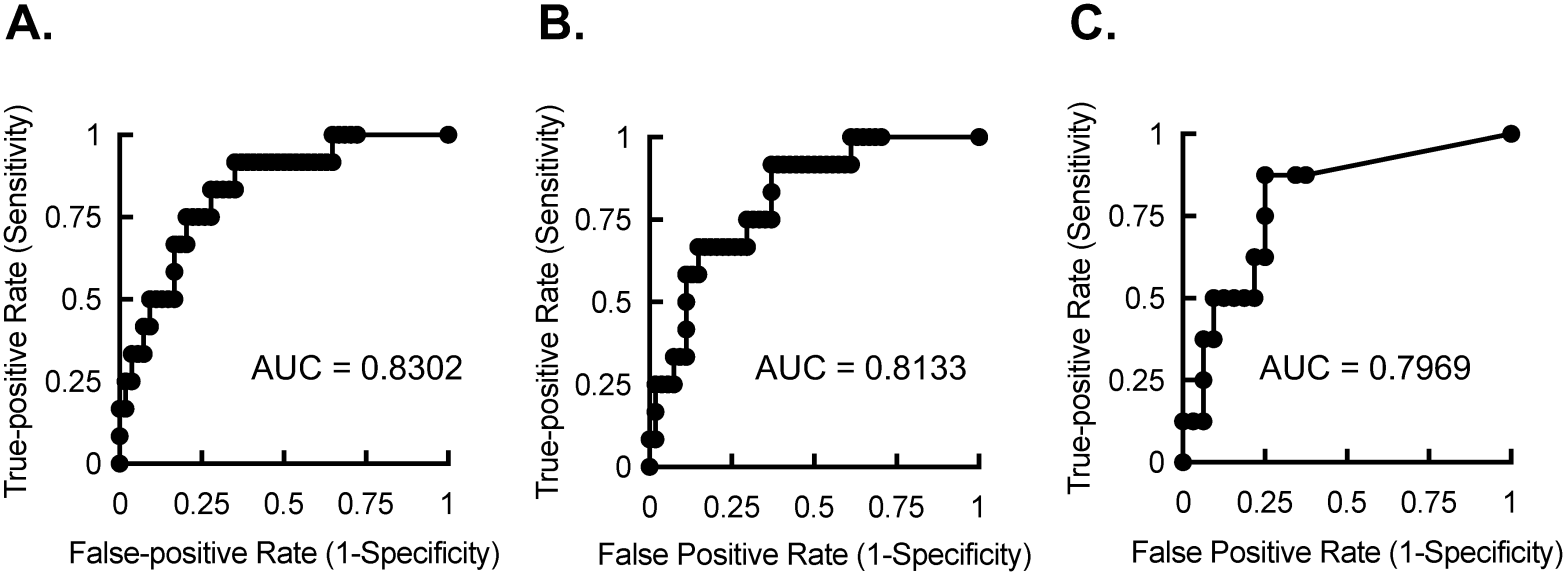
Receiver operator characteristic curves for CCT, LCSCT, and ACCT. (A) ROC curve for CCT with AUC of 0.8302 (P = 0.0004). (B) ROC curve for LCSCT with AUC of 0.8133 (P = 0.0007). (C) ROC curve for ACCT with AUC of 0.7969 (P = 0.01).

In our patient population, the median follow-up time for MACE was 24 (IQR: 20, 24) months for LCSCT and CCT, and 24 (IQR: 16, 24) months for ACCT, and there was a total of 12 MACE events; 1 cardiac death, 2 noncardiac deaths, 1 CVA, 2 CABGs and 6 PCIs. Fig 3 demonstrates the receiver operating characteristic (ROC) curves for the three imaging modalities of CCT, LCSCT and ACCT. The AUC for the ROC curves were 0.8302 (95% confidence interval: 0.7112 to 0.9493), 0.8133 (95% confidence interval: 0.6922 to 0.9344), and 0.7969 (95% confidence interval: 0.624 to 0.9698) for CCT, LCSCT, and ACCT, respectively. Comparison of the AUCs revealed no significant differences between LCSCT and CCT (P = 0.691), ACCT and CCT (P = 0.678), or LCSCT and ACCT (P = 0.839). Despite comparable sensitivity and specificity among the three tests, the cutoff values for optimal test performance were quite different. At CACS of >250, the CCT scans had an 83% sensitivity and a 72% specificity with a likelihood ratio of 3 for MACE in this patient population. Comparable performance could be obtained at CACS >200 and CACS >10 for LCSCT and ACCT, respectively.

**Fig 4:**
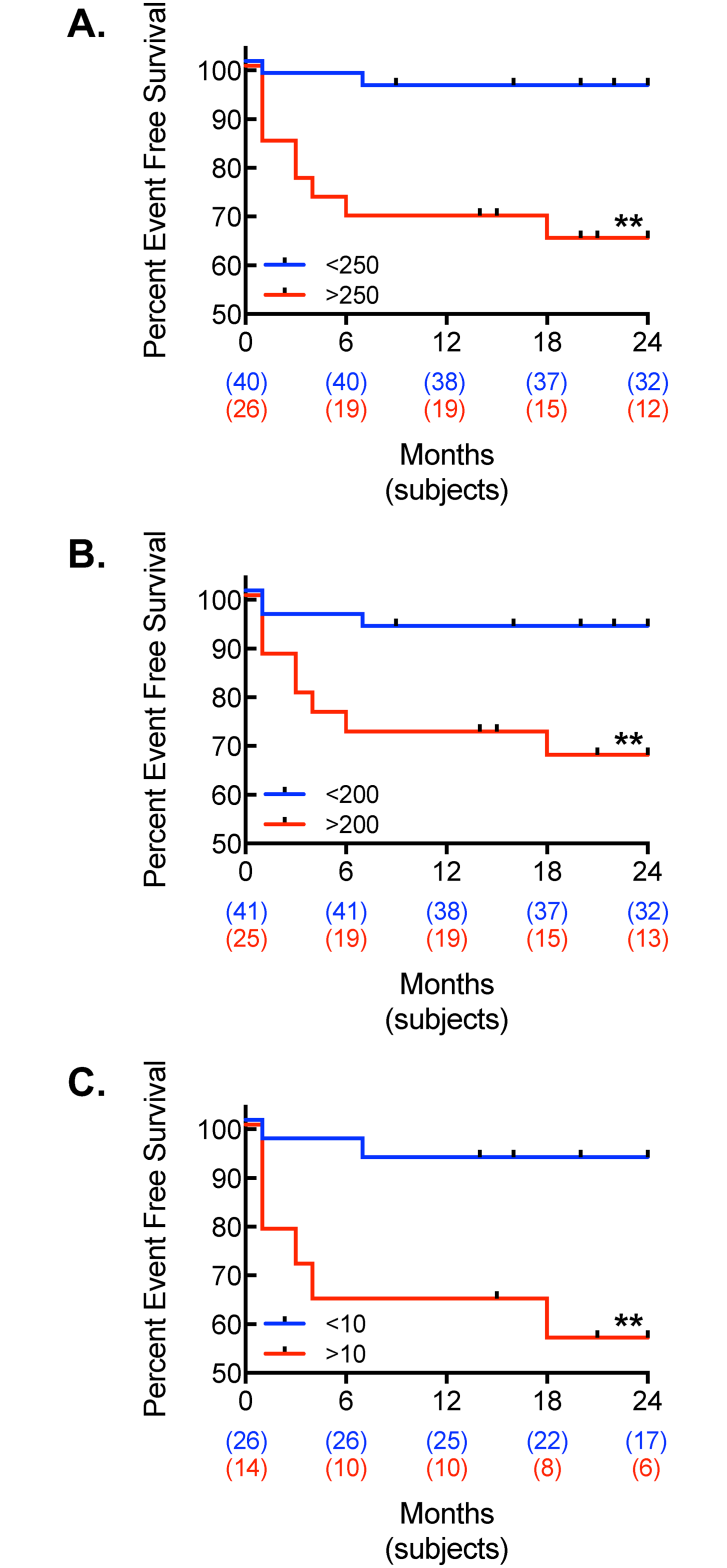
Kaplan-Meier plots for MACE. Kaplan-Meier event curve for MACE using (A) CCT, (B) LCSCT, and (C) ACCT in patients divided into low and high calcium burden as determined by their respective ROC Curves (**, P < 0.01, log-rank test).

Based on the AUC derived from the ROC curves, patients were divided into high and low risk groups, using the above cutoff values for optimal test performance. Fig 4 demonstrates the Kaplan-Meier plots for MACE outcomes (CACS high-risk cutoffs of >250 for CCT, >200 for LCSCT, and >10 for ACCT). The plots for the low-risk vs. high-risk groups were statistically significant in all CT modalities. When the low risk plots were compared between CCT, LCSCT, and ACCT, there were no statistical differences (P = 0.8818) between the CT modalities. Likewise, when the high-risk plots were compared between CCT, LCSCT, and ACCT, there were no differences (P = 0.784) between the CT modalities.

## Discussion

We evaluated the significance of Agatston-derived CACS values derived from LCSCT and ACCT scans in an elevated risk population and found that LCSCT demonstrated better agreement with ECG-gated CCT as the gold standard. Secondary analysis confirmed that no individual vessel was responsible for the lower agreement by ACCT. Though LCSCT and ACCT demonstrated comparable area under the curve (AUC) for the receiver operating characteristic (ROC) curve, cutoff values for elevated risk and optimal test performance revealed LCSCT performs in a manner comparable to CCT. There is robust data to support the use of coronary artery calcium scoring in the further risk stratification of patients with elevated cardiovascular risk[36]. Moreover, a calcium score of zero carried a negative predictive value of 96% for stenosis greater than 50% in a large study of symptomatic patients[37]. However, coronary calcium scoring for precision cardiovascular risk stratification of patients remains underutilized. Furthermore, most insurance providers do not cover the expense of a standard CCT for CACS, and some even consider CCT for CACS to be investigational[31, 36]. In addition to cost, concerns have been raised by studies that have projected a small and finite increase in lifetime cancer risk attributable to CCT, despite technological advances and guidelines that have reduced the radiation exposure below 1 mSv[38, 39]. Thus, demonstrating ability to obtain comparable data regarding coronary calcification from alternative methods may fulfill a large, unmet clinical need. There are an estimated 72 million noncontrast chest CTs done each year in the U.S.[40]. With the recent U.S. Preventative Services Task Force recommendations for lung cancer screening with low-dose computed tomography in adults who have a long-standing history of smoking, the use of LCSCT is expected to grow tremendously; it is estimated that as many as 94 million U.S. adults are current or former smokers[15, 41]. Our current study assessed the utility of reporting CACS from these studies to provide cardiovascular risk assessment and clinical management benefit with negligible added expense or radiation exposure.

We identified CACS values from LCSCT studies as essentially comparable to those from CCT despite the lack of ECG-gating. Recently, LCSCT was determined to underestimate calcium score, particularly in the lower range (<1000) of calcium scores[31]. There are several technical explanations for our study’s improved agreement in the lower range calcium scores. First, by performing our analysis on individual vessel CACS values, we were able to increase the power of our study while focusing on CACS values <800. Second, our study was unique in that we used dedicated lung cancer screening CT scans that had image reconstruction slice thicknesses of 2 mm, which is a comparable thickness to the standard of 2.5 mm ECG-gated cardiac CT scans. The prior study used CACS derived from whole body CT scans with image reconstruction thickness of 6 mm and included a large proportion of CACS values in excess of 1000[31]. Finally, the prior study used CT studies that were performed on an older model electron beam CT scanner between 2000 and 2003, while our study used CT images acquired on a newer 64-slice thickness CT scanner between 2012 and 2015.

Recently, CACS derived from ACCT have demonstrated good agreement, using subjective estimations of calcium burden based on a visual scoring system[27]. Our study differed in that we took a standardized approach to acquisition and scoring that eliminated subjectivity, potential variability among less experienced users, and the increased risk of false positive identification of calcium. By using a more universally applicable and standardized approach, we identified a significantly weaker agreement with a strong negative bias in calcium scores from ACCT studies. Recent studies have sought to change scan acquisition or the thresholding for calcification to <130 HU to make up for the reduced detection of calcium on ACCT scans[28, 29]. Fig 2 illustrates an example of visually appreciable calcium in the LAD that was under-identified because it did not meet the 130 HU threshold for calcium scoring. However, it remains unclear whether reducing the threshold would reduce or sacrifice the specificity of calcium detection, and thus further study is needed in this area.

Coronary artery motion, particularly the motion of the right coronary artery (RCA), during cardiac phases is highly susceptible to imaging artifact in the absence of ECG-gating[42]. However, ECG-gating did not appear to affect the identification of calcium by LCSCT, and results from the individual vessel analysis demonstrated that all four vessels rather than just the RCA contributed to the under identification of calcium on the ACCT studies. Moreover, rotation time and pitch were comparable between LCSCT and ACCT acquisition protocols, indicating that they are not likely to be contributing to the differences in calcium detection. Several alternative explanations for the decreased identification of calcium on ACCT studies remain to be studied. Foremost, the ACCT images were reconstructed at the standard 5 mm slice thickness, whereas the CCT and LCSCT images were reconstructed at 2.5 and 2 mm, respectively. Consistent with the increased slice thickness is the reduced number of slices containing the heart on the ACCT axial images; thus, the under-identification of calcium associated with ACCT may have been influenced by partial volume effects, leading to reduced attenuation measures. Drawing absolute conclusions about image reconstruction thickness from the data is confounded by the other differences in scanning and reconstruction parameters, including collimation, matrix size, and field of view (Table 1). However, field of view and matrix size contribute the minimal pixel area required to identify calcium, and thus it is interesting to note that LCSCT had the largest minimal calcium area of 2.4 mm^2^ whereas ACCT had the smallest minimal area at 1.37 mm^2^, suggesting that these parameters were less likely to contribute to an under-identification of calcium in ACCT studies. Future studies that compare variation in calcium identification conferred by image reconstruction thickness while holding these other acquisition and reconstruction parameters constant are required to understand the extent to which reconstruction thickness impacts calcium score. In addition, we cannot rule out other technical differences from the ACCT scanner, particularly the intrinsic wider collimation, which may also theoretically cause partial volume effects that decrease sensitivity for detection of calcium. Further research is clearly needed to delineate the respective influences of image reconstruction thickness, acquisition parameters of collimation, matrix, and field of view, and scanner type.

We also identified that CACS from LCSCT and CCT had essentially equivalent predictive power for outcomes. This was consistent with other studies evaluating low-dose, noncontrast CT scans in asymptomatic populations[19, 31], however, our study differed in that it was a direct comparison of dedicated LCSCT with CCT in an elevated risk population using comparable image reconstruction thickness. The ROC curves for ACCT and CCT were similar though, as expected, the absolute CACS cutoff values for comparable sensitivity and specificity differed between the imaging approaches. It is possible that our study is underpowered to detect a significant difference in the AUCs between ACCT and CCT, but from a practical perspective there is likely little clinical meaning in the difference between AUCS of 0.8302 and 0.7969. However, the major difference is in the utility of the significantly lowered threshold of optimal test performance for indicating elevated event risk in the ACCT study. Whereas similar risk assessment standards established by CCT can be applied to LCSCT because of the similar test performance and similar absolute CACS values, such standards could not be applied to ACCT without marked reductions in sensitivity and negative predictive value.

There are a number of limitations to this study that must be acknowledged. This was a retrospective study design that took place at a single institution. Our sample size was relatively small and events were primarily driven by revascularization, and so it will be important to confirm these findings in a larger, prospective study designed to include cardiovascular events. Finally, further studies regarding image reconstruction thickness and scanner limitations are required before any final conclusions can be drawn about the potential utility of ACCT derived CACS.

## Conclusions

In summary, CACS by the Agatston method can be readily carried out in LCSCT and ACCT studies. Despite a lack of ECG-gating, the CACS from LCSCT can be considered as essentially comparable to CACs from ECG-gated CCT in terms of absolute value and predictive power, provided image reconstruction thickness and scanner type are similar as they were in this study. CACS from ACCT tended to under-identify coronary calcification. Though ACCT retained a similar AUC for the ROC curve relative to CCT, the cutoff for optimal test performance was markedly lower such that it would be difficult to practically assess CACS in ACCT studies. Thus, it is currently not reasonable to use Agatston-derived CACS values from ACCT images; however, more studies will be required to ultimately define whether alternative assessments of coronary calcification using ACCT can aid in the assessment of cardiovascular risk. Nevertheless, standard reporting of Agatston CACS from LCSCT is accurate and can provide predictive value for MACE, indicating CACS measures should be reported from these studies and factored into an individualized cardiovascular risk assessment.

## Abbreviations

ACCT: Attenuation correction computed tomography
ASCVD: Atherosclerotic cardiovascular disease
AUC: Area under the curve
BMI: Body mass index
CABG: Coronary artery bypass graft surgery
CACS: Coronary artery calcium score
CAD: Coronary artery disease
CCT: ECG-gated cardiac computed tomography
CT: Computed tomography
DM: Diabetes mellitus
ECG: Electrocardiography
HU: Hounsfield Units
keV: Kiloelectron volts
LAD: Left anterior descending
LCSCT: Lung cancer screening computed tomography
LCx: Left circumflex
LM: Left main
MACE: Major adverse cardiac events
mAmp: Milliamperes
MI: Myocardial infarction
PACS: Picture archiving communication system
PCI: Percutaneous intervention
PET: Positron emission tomography
RCA: Right coronary artery
ROC: Receiver operating characteristic
SEE: Standard error of the estimate
SPECT: Single-photon emission computed tomography
VA: Veterans Affairs
